# Papillomatous Lesion of the Right Retromolar Area With Underlying Endothelial Proliferation: A Diagnostic Challenge

**DOI:** 10.1155/crid/1301881

**Published:** 2026-09-11

**Authors:** Javad Sarabadani, Nooshin Mohtasham, Shayan Yousefi, Nazanin Salmani

**Affiliations:** ^1^ Faculty of Dentistry, Mashhad University of Medical Sciences, Mashhad, Iran, mums.ac.ir; ^2^ Patient Safety Research Center, Clinical Research Institute, Mashhad University of Medical Sciences, Mashhad, Iran, mums.ac.ir

**Keywords:** differential diagnosis, hemangioma, oral mucosa, oral pathology, papilloma

## Abstract

**Background:**

The surface appearance of an oral lesion may not accurately indicate the process occurring in the underlying connective tissue. Vascular proliferations covered by papillary epithelial hyperplasia may therefore clinically resemble benign epithelial lesions.

**Case Presentation:**

A 59‐year‐old man with multiple coronary stent placements and ongoing anticoagulant therapy presented with a painless, pedunculated lesion in the right retromolar area. He reported no history of tobacco or alcohol use. The lesion had been noticed approximately 2 weeks earlier and was repeatedly contacted by the adjacent mandibular right third molar. Its exophytic papillomatous surface suggested squamous papilloma or verruca vulgaris. An excisional biopsy was performed using a 980‐nm diode laser, with minimal intraoperative bleeding. Microscopic examination demonstrated papillomatous epithelial hyperplasia overlying numerous endothelial‐lined vascular channels of predominantly capillary caliber, establishing a diagnosis of capillary hemangioma. At the 2‐week follow‐up, the surgical site showed satisfactory mucosal healing without a clinically evident residual lesion.

**Conclusion:**

This case demonstrates that a clinically epithelial‐appearing oral lesion may have an underlying vascular origin. Correlation of the clinical and histopathologic findings is therefore important when evaluating excised papillomatous lesions.

## 1. Introduction

Papillary and verrucous surface patterns occur in several oral conditions and are not specific to a single pathologic process. Benign epithelial lesions, including squamous papilloma and verruca vulgaris, may share their exophytic appearance with reactive, vascular, and neoplastic lesions [1–4]. Although features such as anatomical site, color, consistency, surface texture, and mode of attachment can help narrow the clinical differential diagnosis, the tissue of origin may remain uncertain until microscopic examination is performed.

Hemangiomas, vascular malformations, and intravascular papillary endothelial hyperplasia are among the vascular entities that may enter the differential diagnosis of an oral soft‐tissue mass [5, 6]. Vascular lesions are often expected to display red‐to‐bluish coloration or other recognizable clinical features; however, atypical presentations may resemble nonvascular growths, including pyogenic granuloma and benign epithelial proliferations [7, 8]. This distinction is relevant not only to diagnosis but also to biopsy planning and control of procedural bleeding.

### 1.1. Purpose of the Report

The present report describes a capillary hemangioma in the right retromolar area that clinically appeared as a pedunculated papillomatous lesion. The case is presented to demonstrate how the epithelial surface may obscure an underlying vascular proliferation and to emphasize the value of clinicopathologic correlation in the evaluation of unusual oral lesions.

## 2. Case Presentation

### 2.1. Patient Information

A 59‐year‐old man with a history of multiple coronary stent placements and ongoing anticoagulant therapy presented for evaluation of an oral soft‐tissue lesion. He reported no history of tobacco use or alcohol consumption. The specific anticoagulant agent, dosage, and duration of treatment were not available in the clinical records reviewed for this report.

### 2.2. Clinical Findings

**Figure 1:**
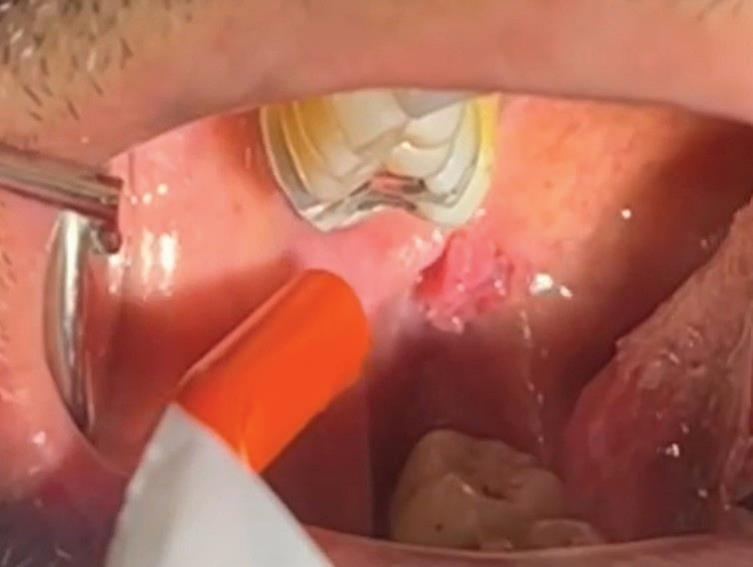
Still frame extracted from the original clinical video showing a soft, pedunculated, exophytic lesion with a papillomatous surface in the right retromolar area.

Clinical examination revealed a peripheral, soft, pedunculated, exophytic lesion with a papillomatous surface in the right retromolar area. The lesion was attached to the mucosa by a thin stalk. The patient had first noticed it approximately 2 weeks before presentation after repeated contact with the adjacent mandibular right third molar drew his attention to the area. He reported no associated pain. The lesion′s clearly peripheral soft‐tissue location and thin pedunculated attachment made a central or intraosseous origin clinically unlikely. Therefore, no dedicated radiographic examination was obtained at the time of presentation (Figure 1).

The clinical appearance and anatomical relationship of the lesion are further demonstrated in Video S1.

### 2.3. Timeline

Approximately 2 weeks before presentation, the patient first noticed the lesion following repeated contact with the adjacent mandibular right third molar. At the initial clinical visit, the lesion was examined, and squamous papilloma and verruca vulgaris were considered the leading differential diagnoses. An excisional biopsy was subsequently performed using a 980‐nm diode laser. At the 2‐week postoperative review, the excision site demonstrated satisfactory mucosal healing without a clinically evident residual lesion, and extraction of the adjacent third molar was recommended to prevent further trauma.

### 2.4. Differential Diagnosis (Clinical)

On clinical examination, squamous papilloma and verruca vulgaris were considered the leading diagnostic possibilities because of the lesion′s pedunculated attachment and papillary surface. A vascular lesion was not initially favored because the lesion lacked conspicuous red‐to‐bluish coloration and otherwise resembled a benign epithelial growth.

### 2.5. Intervention

**Figure 2:**
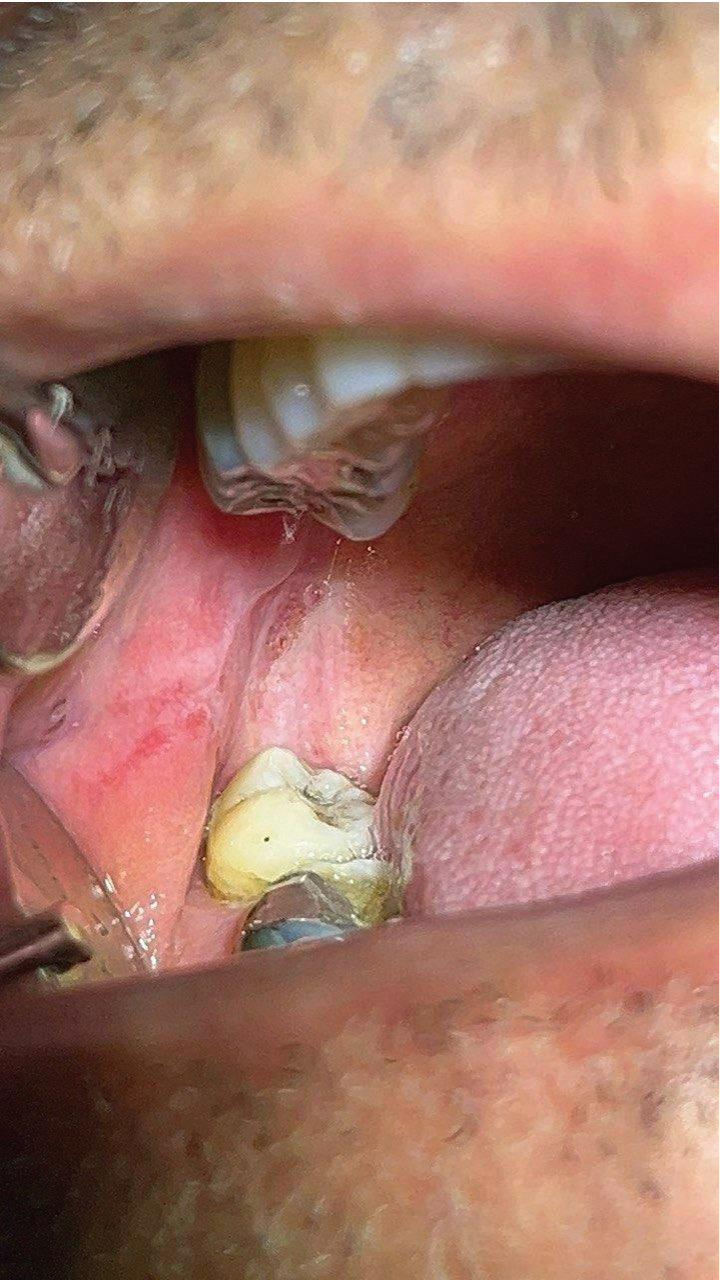
A 2‐week postoperative photograph showing satisfactory mucosal healing of the excision site in the right retromolar area, with mild residual erythema and no clinically evident residual lesion.

Given the patient′s history of multiple coronary stent placements and ongoing anticoagulant therapy, a surgical technique permitting controlled soft‐tissue excision and effective intraoperative hemostasis was preferred. An excisional biopsy was performed using a 980‐nm diode laser (Doctor Smile, Lambda S.p.A., Vicenza, Italy) operated at 1.5 W in continuous‐wave mode, in contact with the tissue through a 600‐*μ*m surgical fiber, with a reported energy density of 6 J/cm^2^. The diode laser was selected to facilitate precise soft‐tissue excision and intraoperative hemostasis, particularly because the patient was receiving anticoagulant therapy. The excised tissue was submitted for histopathologic examination. Intraoperative bleeding was minimal and readily controlled. At the 2‐week follow‐up, the excision site demonstrated satisfactory mucosal healing, with no clinically evident residual lesion (Figure 2). Extraction of the adjacent mandibular right third molar was subsequently recommended to prevent further local trauma.

### 2.6. Histopathologic Findings

The received specimen consisted of six soft, cream‐colored tissue fragments with a total combined size of approximately 0.8 × 0.5 × 0.3 cm.

**Figure 3:**
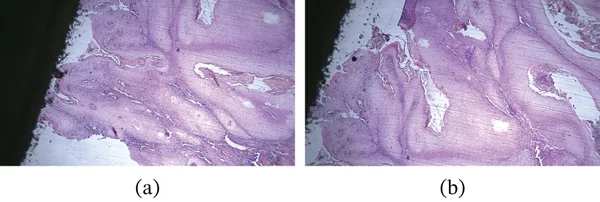
(a,b) Histopathologic section showing papillomatous hyperplasia of the stratified squamous epithelium overlying a vascular connective‐tissue proliferation (hematoxylin and eosin stain).

**Figure 4:**
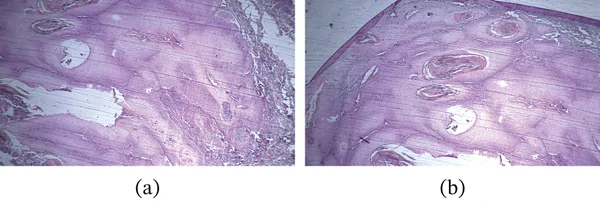
(a,b) Histopathologic section showing numerous capillary‐sized vascular channels of variable caliber with well‐defined endothelial lining in the underlying connective tissue (hematoxylin and eosin stain).

Microscopic examination showed hyperplastic stratified squamous epithelium with a papillomatous architecture and focal hyperparakeratosis. The underlying connective tissue contained numerous endothelial‐lined vascular channels of predominantly capillary caliber and variable size. A mild focal lymphoplasmacytic inflammatory infiltrate was also observed (Figures 3 and 4). Based on these findings, the histopathologic diagnosis was capillary hemangioma. The corresponding histopathology report is provided as File S1. The microscopic findings demonstrated the discrepancy between the lesion′s clinically papillomatous appearance and its underlying vascular nature.

## 3. Discussion

A papillary or verrucous surface pattern is not specific to a single oral disease. Although such morphology is frequently associated with benign epithelial proliferations, similar appearances may occur in reactive, infectious, vascular, and neoplastic processes. Accordingly, lesions such as verruca vulgaris, verruciform xanthoma, pyogenic granuloma, verrucous carcinoma, and papillary squamous cell carcinoma may enter the clinical differential diagnosis of an exophytic papillomatous growth [1–3]. Distinguishing among these conditions may be especially difficult when the lesion develops at an uncommon site or does not display its expected clinical characteristics.

Hemangiomas, vascular malformations, and intravascular papillary endothelial hyperplasia represent distinct vascular abnormalities of the oral cavity. Hemangiomas are commonly described as soft or compressible lesions with red‐to‐bluish coloration, whereas intravascular papillary endothelial hyperplasia, also known as Masson′s tumor, is a reactive endothelial process that develops within a vascular space [9, 10]. However, the vascular origin of a lesion may be concealed when endothelial proliferation is covered by or associated with prominent epithelial changes [5, 6, 11]. In these situations, the surface morphology may direct the clinician toward an epithelial diagnosis despite the presence of an underlying vascular component.

The present lesion illustrates this diagnostic mismatch. Clinically, it appeared as a soft, pedunculated, exophytic growth with a papillomatous surface in the right retromolar area. Its narrow stalk and absence of conspicuous red‐to‐bluish coloration were more suggestive of squamous papilloma or verruca vulgaris than of a vascular lesion. Microscopic examination, however, showed numerous endothelial‐lined vascular channels of predominantly capillary caliber beneath hyperplastic stratified squamous epithelium with a papillomatous configuration. The histopathologic findings therefore established the diagnosis of capillary hemangioma and demonstrated that an atypically presenting oral vascular lesion may not be recognizable from clinical inspection alone [12].

Repeated contact with the adjacent mandibular right third molar may have influenced the surface characteristics of the lesion. Mechanical irritation could have contributed to the epithelial hyperplasia or made the lesion more noticeable to the patient. Nevertheless, the available evidence does not establish trauma as the cause of the vascular proliferation. Its role should therefore be interpreted as a possible modifying factor rather than a confirmed etiologic mechanism.

The patient′s cardiovascular history and ongoing anticoagulant therapy were also relevant to treatment planning. A 980‐nm diode laser was selected to allow controlled excision while facilitating intraoperative hemostasis. Bleeding was minimal and readily controlled, and the surgical site showed satisfactory mucosal healing without a clinically evident residual lesion at the 2‐week review. Favorable hemostatic and postoperative outcomes have likewise been reported after laser treatment of appropriately selected small oral capillary hemangiomas [13]. When a 980‐nm diode laser is used, treatment parameters—including power, emission mode, fiber diameter, tissue contact, and exposure time—should be selected carefully to balance effective cutting and coagulation against the risk of excessive thermal injury [14]. The choice of treatment should therefore be individualized according to the lesion′s size, depth, anatomical site, suspected diagnosis, and the patient′s medical condition.

### 3.1. Strengths and Limitations

The value of this report lies in the documentation of a clinically papillomatous lesion that was ultimately diagnosed histopathologically as a capillary hemangioma. The case brings together the patient′s cardiovascular history and anticoagulant use, the lesion′s retromolar location and unusual morphology, the possible influence of local mechanical irritation, the microscopic findings, and the rationale for diode‐laser excision. The diagnosis was established through examination of the original histologic sections by an oral pathologist. Detailed laser parameters, a supporting preoperative clinical video, and a 2‐week postoperative photograph provide additional documentation of the diagnostic and therapeutic course.

Several limitations must also be considered. No dedicated radiographic examination was performed. Because the lesion appeared confined to the superficial soft tissue and was attached by a narrow pedunculated base, a central or intraosseous origin was considered clinically unlikely. Nevertheless, imaging would have allowed a more objective assessment of the adjacent bone and possible deeper involvement. Imaging is particularly relevant when the clinical findings raise concern for an intraosseous vascular process [11]. In addition, no separate high‐resolution preoperative photograph was obtained because the lesion was documented primarily by video. Figure 1 was therefore created from the clearest available video frame, whereas the complete recording has been retained as supporting information.

Additional high‐resolution histopathologic microphotographs could not be retrieved from the pathology center. The included images are therefore the clearest available representative microphotographs and were prepared at 300 dpi for publication. Their limited ability to display every microscopic diagnostic feature should be distinguished from the diagnostic assessment itself, which was based on examination of the original histologic sections. The clinical records also did not provide the name, dosage, or duration of the patient′s anticoagulant therapy. Furthermore, follow‐up was limited to 2 weeks; although early mucosal healing was documented, long‐term stability and recurrence could not be evaluated. Finally, observations from a single case cannot be generalized to all papillomatous oral lesions, and no causal relationship can be established between repeated mechanical trauma and either the epithelial or vascular changes.

This case demonstrates that the visible surface of an oral lesion may not accurately indicate its underlying tissue of origin. A clinically benign papillomatous growth may conceal a vascular proliferation, particularly when typical vascular features are absent. Histopathologic evaluation of excised tissue is therefore necessary when the clinical appearance alone does not permit a confident diagnosis.

## 4. Conclusion

A pedunculated oral lesion with a papillomatous surface may occasionally represent a vascular rather than a purely epithelial process. In this case, histopathologic examination identified capillary‐sized endothelial‐lined channels beneath the papillomatous epithelium and established the diagnosis of capillary hemangioma. The findings support careful correlation of the clinical appearance, patient history, and microscopic features when evaluating unusual oral soft‐tissue lesions.

## Author Contributions

Javad Sarabadani: conceptualization, investigation, resources, supervision, project administration. Nooshin Mohtasham: investigation, validation, resources, supervision. Shayan Yousefi: investigation, data curation, writing—original draft preparation. Nazanin Salmani: conceptualization, data curation, visualization, project administration, writing—original draft preparation, writing—review and editing.

## Funding

No funding was received for this manuscript.

## Disclosure

All authors have read and approved the final version of the manuscript. Nazanin Salmani, the corresponding author and manuscript guarantor, had full access to all of the data in this study and takes complete responsibility for the integrity of the data and the accuracy of the data analysis.

## Ethics Statement

This study did not require formal ethical approval according to institutional guidelines for single anonymized case reports. All procedures were performed as part of routine clinical care. Written informed consent was obtained from the patient for publication of the clinical details, images, and supporting video.

## Consent

All the patients allowed personal data processing and informed consent was obtained from all individual participants included in the study.

## Conflicts of Interest

The authors declare no conflicts of interest.

## Supporting Information

Additional supporting information can be found online in the Supporting Information section.

## Supporting information


**Supporting Information 1** File S1: Histopathology report confirming the diagnosis of capillary hemangioma.

**Supporting Information 2** Video S1: Clinical video demonstrating the exophytic, papillomatous lesion.

**Supporting Information 3**  

## Data Availability

The data that support the findings of this study are available in the supplementary material of this article.
