# Supplementary material for: Papillomatous Lesion of the Right Retromolar Area With Underlying Endothelial Proliferation: A Diagnostic Challenge
Source: Case Rep Dent. 2026 Sep 11;2026:1301881. doi: 10.1155/crid/1301881 (PMC13563060; doi:10.1155/crid/1301881)
Supplement: Supplementary file 2 [file CRID-2026-1301881-s002.docx]

**Macroscopic Description:**

The received specimen consisted of six soft, cream-colored tissue fragments, with a total combined size of approximately 0.8 × 0.5 × 0.3 cm.

**Microscopic Findings:**

Microscopic examination of the submitted specimen revealed proliferation of blood and lymphatic vessels in the underlying connective tissue (chorion), forming numerous capillaries of varying calibers with well-defined endothelial lining. Scattered areas showed mild lymphoplasmacytic infiltration. The lesion’s surface was covered by hyperplastic stratified squamous epithelium with a papillomatous architecture and focal hyper parakeratosis.

**Diagnosis:**

Oral mucosa biopsy:

- Capillary hemangioma

**Dr.NooshinMohtasham**

MD AP-AC

Consultant Oral Pathologist
